# Delayed Onset of Bullous Pemphigoid Secondary to Nivolumab

**DOI:** 10.7759/cureus.43230

**Published:** 2023-08-09

**Authors:** Eric Olsen, Steven A Svoboda, Sami K Saikaly, Tricia A Missall, Kiran Motaparthi

**Affiliations:** 1 Department of Dermatology, University of Michigan Medical School, Ann Arbor, USA; 2 Department of Dermatology, University of Florida, Gainesville, USA

**Keywords:** nivolumab, immunotherapy, bullous pemphigoid, drug-induced bullous pemphigoid, cancer immunotherapy, treatment choices, immunotherapy-related adverse events

## Abstract

The increasing use of immune checkpoint inhibitors, such as nivolumab, a programmed cell death protein 1 (PD-1) inhibitor, for advanced neoplastic disease has revealed significant cutaneous immune-related adverse effects. Herein, we report a case of bullous pemphigoid (BP) secondary to nivolumab therapy for recurrent metastatic oropharyngeal squamous cell carcinoma. In this patient, the time to development of BP was three years, which represents the most delayed onset of BP secondary to a PD-1 inhibitor that has been reported in the literature. Symptoms were initially controlled on low-dose oral prednisone but recurred after two years. The patient was subsequently treated with a several-month taper of high-dose oral prednisone, during which he was able to resume nivolumab without recurrence of skin lesions. Although immune checkpoint inhibitor-induced BP remains rare, physicians should be aware of this serious cutaneous immune-related adverse event as the use of this drug class continues to expand.

## Introduction

Immune checkpoint inhibitors (ICIs), such as programmed cell death protein 1 (PD-1) and programmed death-ligand 1 (PD-L1) inhibitors, are monoclonal antibodies that are increasingly being utilized to treat a growing number of solid and hematologic malignancies, including recurrent or metastatic squamous cell carcinoma of the head and neck, non-small cell lung carcinoma, advanced renal cell carcinoma, urothelial carcinoma, and melanoma [1]. The PD-1/PD-L1 pathway normally functions to stop or limit the development of the T cell response via the interaction of PD-1 on activated T cells and PD-L1 and PD-L2 on dendritic cells or macrophages [2]. This interaction ensures appropriate activation of the T cell response to prevent chronic autoimmune dysregulation. Many tumor cell types exploit the PD-1/PD-L1 pathway via overexpression of PD-L1 to evade the body’s adaptive immune response [2]. Thus, by preventing tumor-expressed PD-L1 from binding to PD-1 receptors on activated T cells, PD-1/PD-L1 inhibitors enable cytotoxic T cells to effectively target tumor cells [1].

As a consequence of their immunoregulatory mechanism of action, PD-1/PD-L1 inhibitors commonly cause various immune-related adverse events (irAEs), including those related to the skin [3]. Tang et al. reported that cutaneous irAEs occur in 20-40% of patients treated with PD1/PD-L1 inhibitors and most frequently present as lichenoid reactions, eczematous reactions, or vitiligo [4]. More rare skin manifestations that have been reported include morbilliform eruptions, bullous pemphigoid (BP), and Stevens-Johnson syndrome/toxic epidermal necrolysis (SJS/TEN) [3]. In particular, BP secondary to PD-1/PD-L1 inhibition is exceedingly rare. A systematic review from August 2022 noted 127 cases of ICI-related BP documented in the literature [5]. The reported median time to onset of BP following initiation of ICI therapy is six months, and the longest reported latency period is two years [6].

BP is an autoimmune blistering disorder that clinically presents as pruritic urticarial plaques with overlying tense bullae [7]. The development of subepidermal blisters occurs in response to autoantibodies targeting the hemidesmosomal proteins BP180 (BP antigen 2, collagen XVII) and BP230 (BP antigen 1) at the dermal-epidermal junction [7]. Development of BP has been traditionally associated with increased age, neurological disease, and internal malignancy. The use of various pharmacologic agents has also been associated with BP, including loop diuretics and dipeptidyl peptidase-4 inhibitors [8].

The case reported herein represents the most delayed presentation of BP secondary to a PD-1 inhibitor, to the best of our knowledge. Our patient developed BP after three years on nivolumab for the treatment of his recurrent metastatic oropharyngeal squamous cell carcinoma.

## Case presentation

A 61-year-old Caucasian male presented to the dermatology clinic in September 2022 with one month of a pruritic blistering rash on the trunk and extremities. His medical history was significant for recurrent metastatic oropharyngeal squamous cell carcinoma, which was diagnosed in November 2015 and initially treated with external beam radiation therapy (XRT) and carboplatin/docetaxel. In April 2017, tumor progression led to the initiation of nivolumab 3 mg/kg IV every two weeks, with which the patient was able to achieve stable control of his neoplastic disease. In May 2020, he developed a diffuse pruritic blistering rash on his right lower back and right upper extremity, which resolved with the cessation of nivolumab by medical oncology. At this time, the patient did not receive an evaluation by dermatology or have a diagnosis of BP confirmed by biopsy or serologic testing. The patient’s other medications included omeprazole, aspirin, pilocarpine, lactulose, hydroxyzine, duloxetine, and gabapentin, all of which had remained unchanged since the initiation of nivolumab. In September 2020, medical oncology resumed nivolumab after imaging revealed tumor progression. They also placed the patient on oral prednisone ranging from 5 mg to 15 mg daily to prevent breakthrough rash. The patient remained largely asymptomatic until August 2022 when he experienced a flare in the bullous rash and was subsequently referred to dermatology.

**Figure 1:**
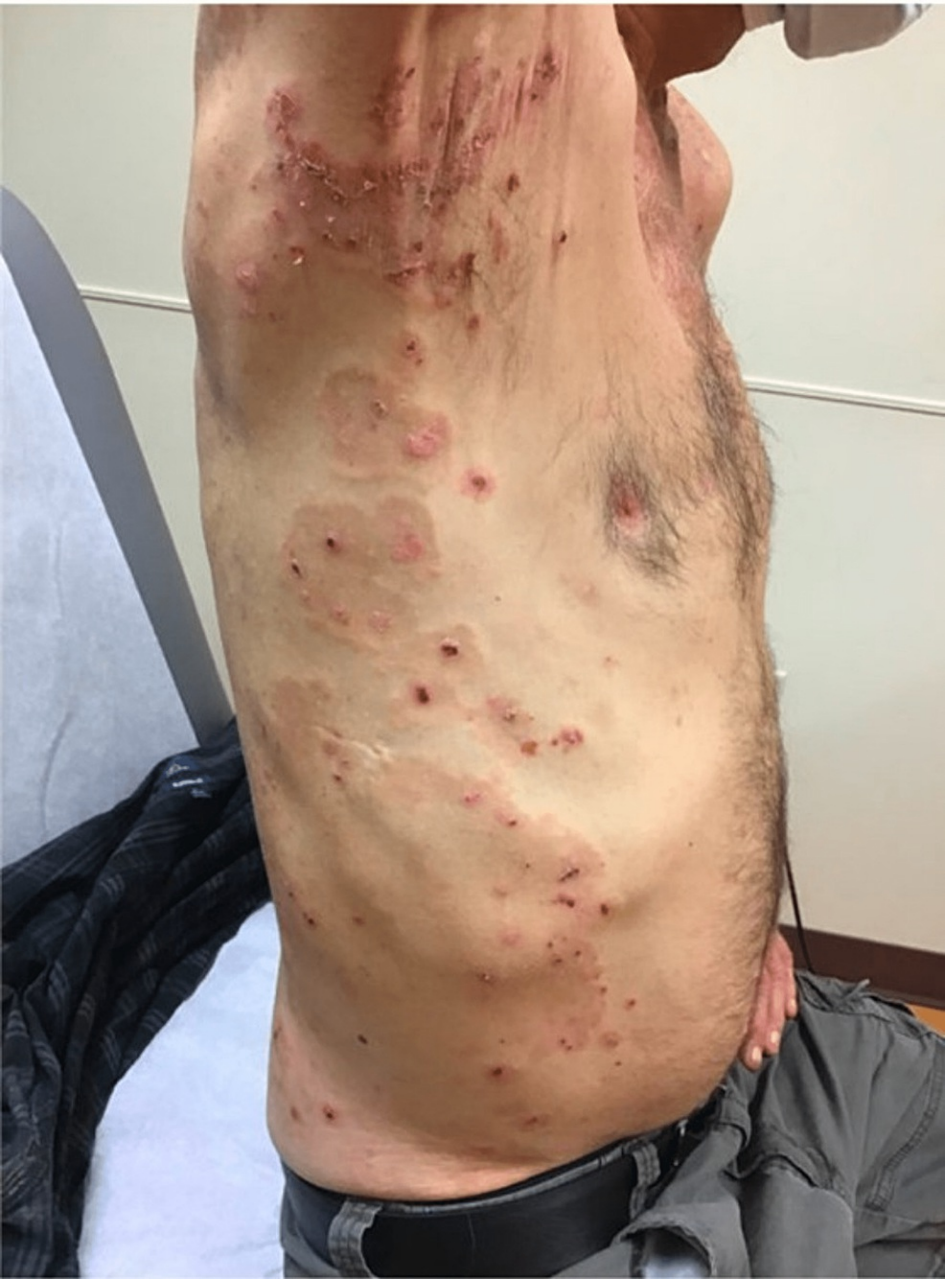
Tense bullae overlying erythematous urticarial plaques on the trunk consistent with the rash of bullous pemphigoid.

**Figure 2:**
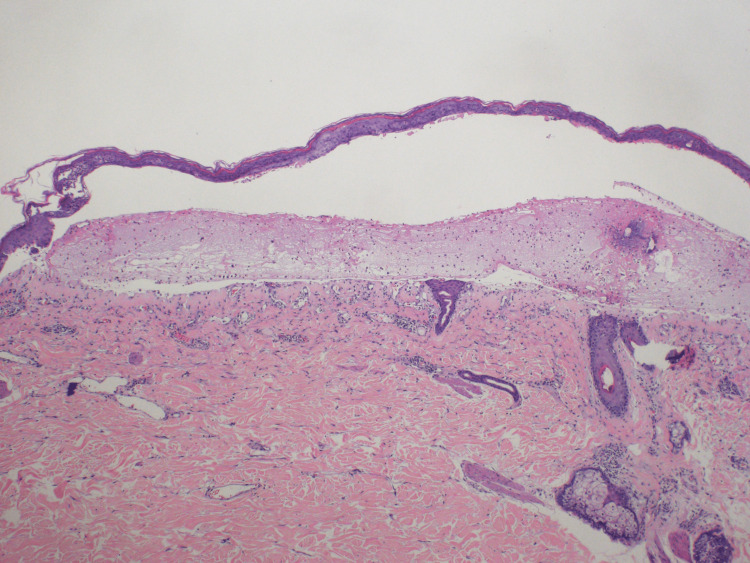
Hematoxylin & eosin stain demonstrating subepidermal vesicle formation.

**Figure 3:**
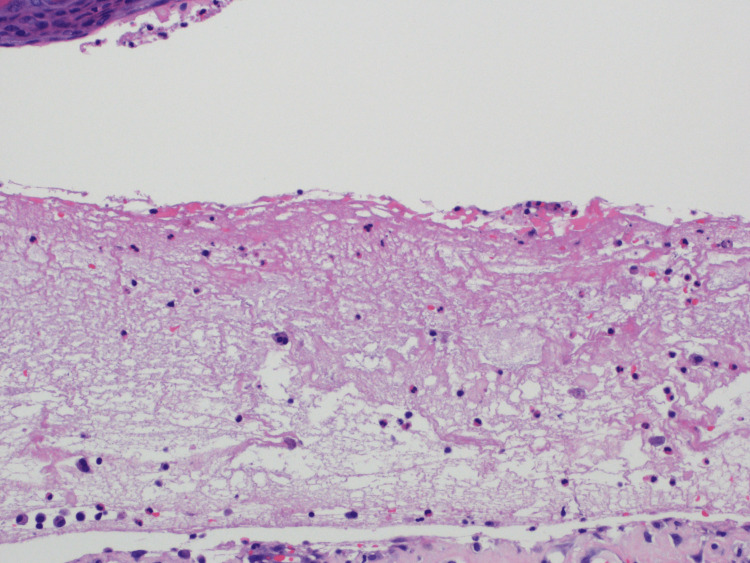
Hematoxylin & eosin stain demonstrating dermal edema with superficial lymphocytic and eosinophilic infiltration.

On examination, the patient exhibited erythematous urticarial plaques with overlying tense bullae, some of which were intact and some with hemorrhagic crust scattered diffusely on the trunk and all extremities (Figure 1). Punch biopsies revealed a subepidermal vesicle arising from the spongiotic epidermis, as well as scattered eosinophils and neutrophils within the vesicle cavity (Figure 2). Dermal edema with superficial lymphocytic inflammation admixed with numerous eosinophils was observed (Figure 3). Direct immunofluorescence antibody localization demonstrated positive immunoreactivity for IgG and complement C3 in a linear pattern along the basement membrane with negative immunoreactivity for IgA and IgM, which confirmed the diagnosis of BP.

**Figure 4:**
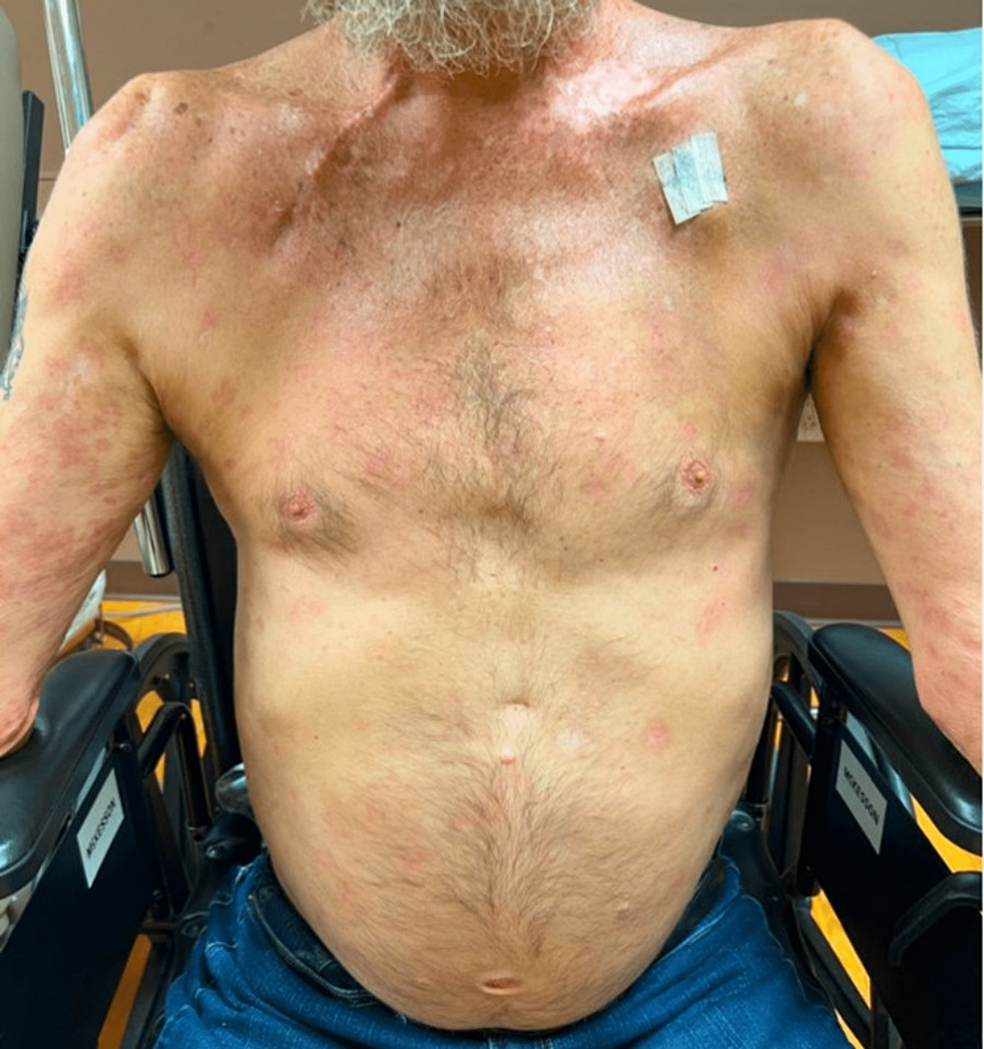
Resolved bullous pemphigoid rash with residual post-inflammatory hyperpigmentation status-post prednisone taper.

**Figure 5:**
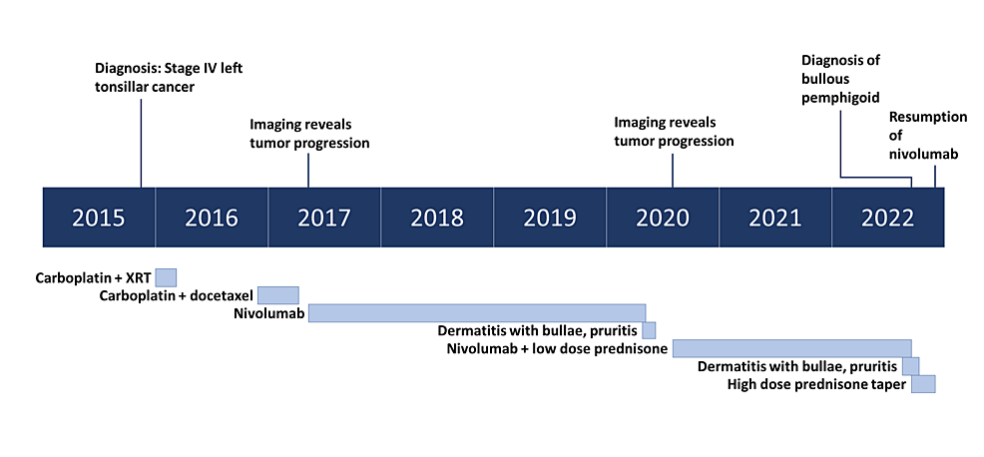
Timeline of key clinical events. XRT: external beam radiation therapy.

The patient was started on oral prednisone 60 mg daily and topical clobetasol ointment by dermatology. Nivolumab was held by medical oncology. This resulted in rapid resolution of the rash within four weeks of initiating treatment, which allowed for subsequent long-term prednisone taper and resumption of nivolumab three months later at the same dose and frequency without recurrence of the BP (Figure 4). Low-dose prednisone was then resumed to prevent recurrence. A timeline is included to illustrate key clinical milestones (Figure 5).

## Discussion

With the emerging widespread use of PD-1/PD-L1 inhibitors in the treatment of numerous oncologic conditions, BP secondary to these agents is increasingly being reported [5]. Our patient detailed above was initiated on nivolumab in April 2017 and noted the development of pruritic bullae in May 2020, 37 months after initial exposure to nivolumab. It is likely that his dermatologic signs and symptoms at this time represented BP, which was controlled for two years with low-moderate dose prednisone maintenance therapy and temporary discontinuation of immunotherapy. The generalized distribution of findings suggests that this is not radiation-induced BP, which is typically confined to the area of radiation exposure. Unfortunately, he did not receive a formal diagnosis of BP until his rash recurred, and he was evaluated by dermatology in September 2022, five years after nivolumab initiation. Nevertheless, this case represents the longest delay in the presentation of BP reported in the literature to date, surpassing a case of BP in a patient with metastatic non-small cell lung cancer that had a latency period of two years [6].

Treatment for ICI-induced BP is dependent on the severity of the disease. For mild or limited BP, the application of high-potency topical corticosteroids twice daily is often sufficient to resolve symptoms [3]. In cases with more extensive skin involvement, such as in our patient, treatment with a short course of high-dose oral corticosteroids followed by long-term therapy with a biologic medication such as rituximab, dupilumab, or omalizumab is warranted. Checkpoint inhibitor treatment should not be interrupted [9]. As corticosteroids attenuate the antitumor response of checkpoint inhibitor therapy and may negatively affect overall survival in patients with neoplastic disease, it is especially important to keep their use brief [10]. Immunosuppressive medications such as mycophenolate mofetil, azathioprine, and methotrexate can also be trialed, as they have been demonstrated to be effective adjunctive or maintenance therapies for BP; however, they have not been studied specifically in patients with ICI-induced BP [11-13].

The monoclonal anti-CD20 antibody rituximab has demonstrated efficacy in the treatment of recalcitrant BP and is regarded as the next step in therapy for severe ICI-induced BP [11,13]. In a review of 10 cases of ICI-induced BP treated with rituximab, all 10 patients had achieved complete resolution [13]. One case report documented complete remission in a patient with nivolumab-induced BP after receiving rituximab 375 mg/m2 once weekly for four weeks with a durable response seen after eight months of follow-up [14]. The proposed mechanistic rationale supporting the use of rituximab in ICI-induced BP is that PD-1 inhibition may enhance B cell antibody formation against BP antigens via dysfunction of B cell regulatory T cells, resulting in disinhibition of B cell autoimmunity [15].

Limited data also support the use of monoclonal antibodies, dupilumab, and omalizumab for treatment-resistant BP. A multicenter case series reported satisfactory responses in 12 out of 13 patients with BP treated with dupilumab, the interleukin-4 receptor antagonist [16]. Several case reports have also documented the effectiveness of dupilumab, specifically in PD-1/PD-L1-associated BP with dosing similar to that for atopic dermatitis, namely, an initial loading dose of 600 mg followed by a maintenance dose of 300 mg every other week [17,18]. For patients with refractory BP treated with omalizumab, the immunoglobulin E (IgE) monoclonal antibody, a systematic review of 56 patients reported an 87% overall response rate (55% complete response and 32% partial response) [19]. The most common dosing of omalizumab for these patients was 300 mg every two to four weeks, which was well tolerated [19]. In regards to ICI-induced BP, omalizumab demonstrated significant improvement in a patient with pembrolizumab-induced BP refractory to prednisolone and intravenous immunoglobulin [20]. Notably, the patient was able to resume pembrolizumab while maintaining complete resolution of BP with a combination of omalizumab and prednisolone. Despite the good response rates and safety profiles of dupilumab and omalizumab, continuous treatment may be required as recurrence is often seen within months of drug cessation [16].

Overall, robust clinical data supporting the use of rituximab, dupilumab, and omalizumab for severe or recalcitrant ICI-induced BP are limited; however, the preliminary findings from case reports and series are promising and warrant further investigation. Furthermore, treatment with these selective monoclonal antibodies for patients receiving life-saving immunotherapy may be a more prudent strategy given that the antineoplastic efficacy of ICIs relies on a strong host T cell response, which may potentially be blunted by traditional global immunosuppressive therapies [1,2]. Future long-term follow-up data will be required to elucidate whether these targeted treatment approaches for severe or recalcitrant BP result in improved outcomes for patients with immunotherapy-responsive oncologic conditions.

## Conclusions

PD-1/PD-L1 inhibitors are effective therapies for numerous malignancies. As the use of PD-1/PD-L1 inhibitors becomes more widespread for the treatment of a growing number of advanced neoplastic conditions, the prevalence of BP secondary to this class of medication will only continue to rise. Therefore, it is critical for physicians to recognize BP as one of the severe cutaneous adverse effects of ICIs, as well as understand how to manage BP in the context of oncologic disease amenable to immunotherapy. Numerous options for the management of immunotherapy-induced BP exist, although systemic corticosteroids remain a mainstay of treatment.
